# Investigation of in-phase bilateral exercise effects on corticospinal plasticity in relapsing remitting multiple sclerosis: A registered report single-case concurrent multiple baseline design across five subjects

**DOI:** 10.1371/journal.pone.0272114

**Published:** 2023-03-02

**Authors:** Dimitris Sokratous, Charalambos C. Charalambous, Eleni Zamba Papanicolaou, Kyriaki Michailidou, Nikos Konstantinou

**Affiliations:** 1 Faculty of Health Sciences, Department of Rehabilitation Sciences, Cyprus University of Technology, Limassol, Cyprus; 2 Physiotherapy Unit, Neurology Clinics, The Cyprus Institute of Neurology and Genetics, Nicosia, Cyprus; 3 Department of Basic and Clinical Sciences, Medical School, University of Nicosia, Nicosia, Cyprus; 4 Centre for Neuroscience and Integrative Brain Research (CENIBRE), University of Nicosia Medical School, Nicosia, Cyprus; 5 Neuroepidemiology Department, The Cyprus Institute of Neurology and Genetics, Nicosia, Cyprus; 6 Biostatistics Unit, The Cyprus Institute of Neurology and Genetics, Nicosia, Cyprus; Faculty of Medicine, Al-Azhar University, EGYPT

## Abstract

Relapsing-remitting Multiple Sclerosis is the most common demyelinating neurodegenerative disease and is characterized by periods of relapses and generation of various motor symptoms. These symptoms are associated with the corticospinal tract integrity, which is quantified by means of corticospinal plasticity which can be probed via transcranial magnetic stimulation and assessed with corticospinal excitability measures. Several factors, such as exercise and interlimb coordination, can influence corticospinal plasticity. Previous work in healthy and in chronic stroke survivors showed that the greatest improvement in corticospinal plasticity occurred during in-phase bilateral exercises of the upper limbs. During in-phase bilateral movement, both upper limbs are moving simultaneously, activating the same muscle groups and triggering the same brain region respectively. Altered corticospinal plasticity due to bilateral cortical lesions is common in MS, yet, the impact of these type of exercises in this cohort is unclear. The aim of this concurrent multiple baseline design study is to investigate the effects of in-phase bilateral exercises on corticospinal plasticity and on clinical measures using transcranial magnetic stimulation and standardized clinical assessment in five people with relapsing-remitting MS. The intervention protocol will last for 12 consecutive weeks (30–60 minutes /session x 3 sessions/week) and include in-phase bilateral movements of the upper limbs, adapted to different sports activities and to functional training. To define functional relation between the intervention and the results on corticospinal plasticity (central motor conduction time, resting motor threshold, motor evoked potential amplitude and latency) and on clinical measures (balance, gait, bilateral hand dexterity and strength, cognitive function), we will perform a visual analysis and if there is a potential sizeable effect, we will perform statistical analysis. A possible effect from our study, will introduce a proof-of-concept for this type of exercise that will be effective during disease progression.

**Trial registration:** ClinicalTrials.gov NCT05367947.

## Introduction

Multiple sclerosis (MS) is the most common inflammatory demyelinating and neurodegenerative disease of the central nervous system [1]. The global prevalence of MS during the last decade has increased by 30%, while the number of people suffering with MS worldwide is estimated at approximately 2.8 million [2]. The low mean age of diagnosis (i.e., 32 years old), along with an average of seven years’ shorter life expectancy (i.e., 74.7 years) compared to the general population [3–5], highlights the need for a lengthy support, resulting in increased financial burden [6]. Recent studies reported that the annual mean cost of health care systems for people with MS living in Europe is about €40,000 [2]. Additionally, both MS patients and their caregivers, who usually are family members, face several psychological and social difficulties due to social isolation, poor quality of life, reduced productivity and lower general health levels [7,8].

Relapsing-remitting MS (RRMS), is the most common type of MS and is characterised by periods of relapses followed by partial or complete recovery [9]. Inflammatory lesions are commonly found bilaterally in both white and grey matter of the central nervous system [10,11], resulting in diverse clinical condition and symptoms, that include motor and cognitive impairments, visual deficits, depression and fatigue [11–13]. Those symptoms results in significantly low quality of life [14,15], which subsequently causes the need for a lifelong support and management of symptoms for most people with RRMS [16].

Motor symptoms in RRMS are associated with changes in corticospinal tract integrity and neuroplasticity [17–23]. The corticospinal tract is one of the major motor descending pathways providing voluntary motor function in humans [24]. The neuroplasticity of the corticospinal tract, is defined by changes in neuron structure or function, detected either directly from measures of individual neurons or inferred from measures taken across populations of neurons [25], is an essential factor that predicts clinical recovery in the post-relapse stage of people with RRMS [26,27]. Corticospinal plasticity can be probed using Transcranial Magnetic Stimulation (TMS) [28,29] and characterized via corticospinal excitability measures including resting motor threshold, motor-evoked potential (MEP) amplitude and latency, and the central motor conduction time (CMCT) [29]. Motor threshold and MEP amplitude are the hallmark measures of corticospinal excitability in MS [30], whereas the MEP latency and CMCT are temporal measures of the corticospinal excitability [31].

Corticospinal plasticity is exercise-dependent [32,33] and influenced by various factors [34,35], such as aerobic exercise [20,36–38], resistance training [20,38], as well as interlimb coordination [39,40]. Previous studies that assessed corticospinal plasticity using TMS in healthy participants and in chronic stroke survivors, reported that interlimb coordination and especially in-phase bilateral movement has the strongest effect on corticospinal plasticity [41–44]. These effects are thought to be due to the suppression of cortical inhibition [44,45] and the simultaneous activation of homologous representations of the motor cortices, which involves interhemispheric facilitation via transcallosal connection between the primary motor cortex and the supplementary motor area [46,47].

Despite the broad literature on the effects of different types of exercises on the neuroplasticity in people with RRMS [37,48–50], it is unclear whether in-phase bilateral exercises can promote motor related neuroplastic changes in RRMS. In light of evidence that people with RRMS have bilateral cortical lesions [51] which cause bilateral changes of corticospinal tract integrity [21,23], these findings raise the question about the effects of in-phase bilateral exercises on corticospinal plasticity. Such effects would provide strong evidence about whether exercise, in particular in-phase bilateral exercise, can influence the corticospinal plasticity in RRMS.

The aim of this study is to investigate whether a 12-week intervention protocol of in-phase bilateral exercises for the upper limbs, which are adapted to sports activities and to functional training, can significantly affect the corticospinal plasticity and subsequently the individual clinical condition of people with RRMS. Our primary hypothesis is that a significant improvement of corticospinal plasticity will be detected bilaterally as measured with CMCT, caused by the specific intervention protocol which includes in-phase bilateral exercises of the upper limbs, in people with RRMS. We will assess the corticospinal plasticity bilaterally using TMS and calculate corticospinal excitability measures [43]. Visual analysis will be conducted separately for each variable and results will be presented graphically according to the level, trend, and stability, to define functional relationships between the intervention protocol and the corticospinal plasticity. Subsequently, if the visual analysis will indicate a potential sizeable effect, we will perform statistical analysis to estimate the effect of the intervention and randomization tests will be constructed to evaluate statistical significance [52].

Exploratory analyses in Stage 2 will investigate the effects of the specific exercises protocol on resting motor threshold, the MEP amplitude and latency, and on clinical symptoms using clinical assessments (i.e., gait, balance, strength, hand dexterity, cognitive functions, Modified Fatigue Impact Scale) [53].

The study follows a single-case concurrent multiple baseline design across subjects [54], which involves five people with RRMS. The specific design has the advantage to verify the cause-effect inference clearly by the staggered duration through separate baseline phases [55]. Consequently, we assume that possible effects from our study will provide preliminary evidence and proof-of-concept evidence for this type of exercise which can be applied during the disease progression and to existing neurorehabilitation protocols.

## Materials and methods

### Participants

All participants will be recruited from March to October 2023 and evaluated by a neurologist at The Cyprus Institute of Neurology and Genetics. The inclusion criteria include 1) diagnosis of RRMS, 2) Expanded Disability Status Scale score between three and five [56], 3) aged between 30 and 70 years, 4) no relapse within 30 days and 5) Mini Mental State of Examination score between 24 and 30 (no cognitive impairment) [57]. The exclusion criteria include 1) brain metal implants (e.g., titanium skull plates, aneurysm clips) [58], 2) history of any disease affecting the central nervous system other than MS (e.g., stroke, Parkinson`s disease, cerebral palsy), 3) history of cardiovascular disease (e.g., known aneurism, myocardial infarction, hyper/hypotension, heart failure), 4) mental disorders (e.g., depression, schizophrenia, bipolar syndrome), 5) severe orthopaedic disorders (e.g., knee or hip replacement, spondylosurgery, disk herniation, recent bone fracture), 6) pregnancy during the implementation of the study timeline, 7) visual deficit (e.g., optic neuritis, blindness, diplopia, glaucoma, blurred vision), 8) hearing impairments (i.e., deafness), 9) history of epileptic seizures and 10) spasticity level on upper or lower limbs more than 1+ (slight increase in muscle tone) according to Modified Ashworth Scale [59]. Additionally, participants will be advised to continue their usual prescribed medication throughout the study duration, and they will be advised to continue their usual routine and avoid receiving any other exercise program during the study. Furthermore, all participants will read and sign a written informed consent, while all procedures are approved and conducted in accordance with the ethical guidelines of the Cyprus National Bioethics Committee before recruitment.

### Study design

**Fig 1:**
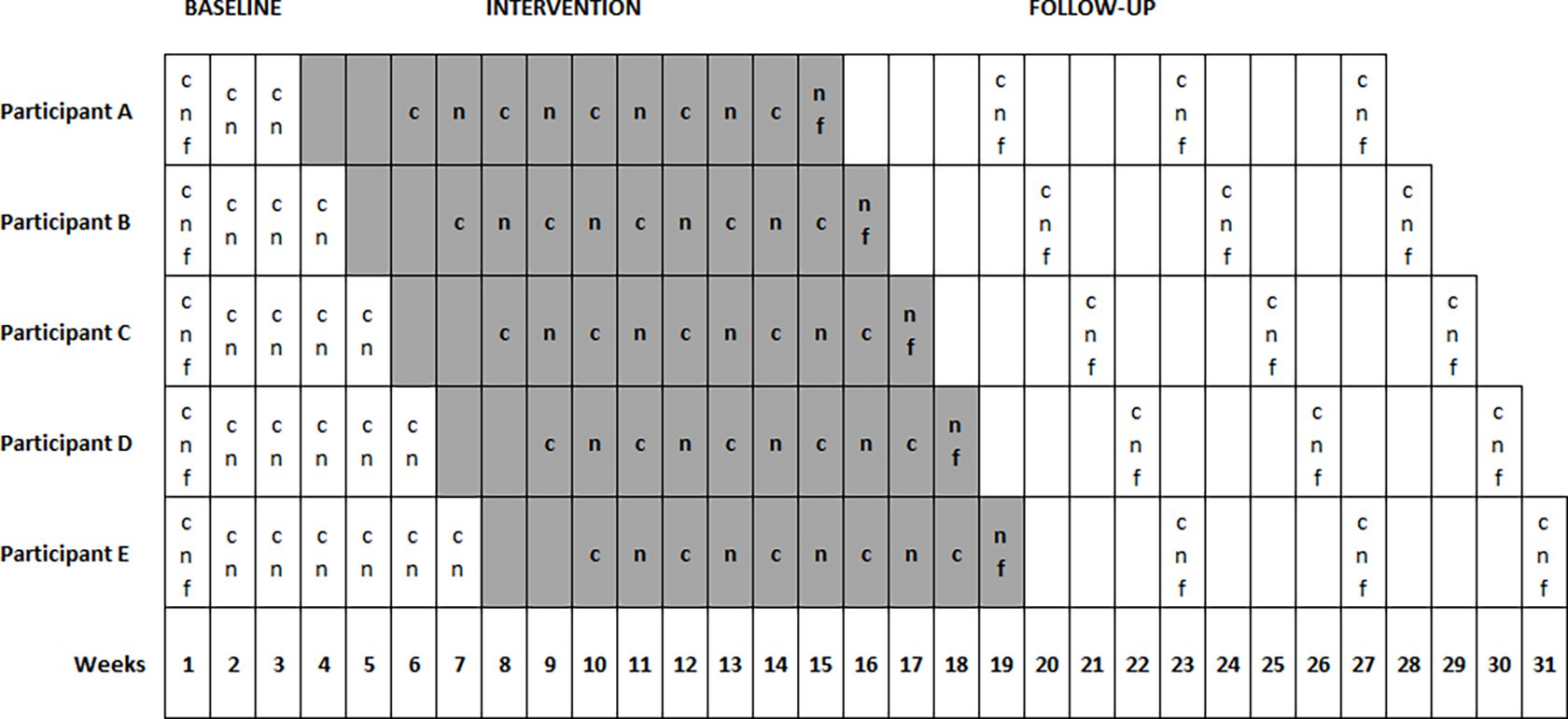
Timeline and schematic representation of the study’s design. Grey colour represents the intervention phase. Each row (A-E) represents a different participant. (c) clinical assessment. (f) Modified Fatigue Impact Scale questionnaire. (n) neurophysiological assessment via TMS. Every cell represents a different week, so every procedure which is included (i.e., c, n, f) will be performed during the corresponding week but in different days.

The specific study is registered on ClinicalTrials.gov, with registration number NCT05367947. The study follows a single-case concurrent multiple baseline design across five subjects, without blinding and has been designed according to the “single case design” criteria, in which three participants [60], each with at least three data points per variable of interest across different phases is the minimum number needed to meet the standard criteria [54]. Therefore, we aim to include five participants to ensure the reliability of the results in case of dropouts, as well as to record several data points across the baseline phase, five data points during the intervention phase and three data points in the follow up phase. During the experimental procedure, all participants will begin the study with the baseline phase at the same time while the intervention phase is introduced staggered across patients and time (Fig 1). The intervention will be introduced systematically in one patient while baseline data collection continues in the others without any intervention. The cause-effect inference can be clearly verified by the staggered duration through separate baseline phases [55]. Subsequently, if the intervention (i.e., in-phase bilateral exercises of the upper limbs) is the sole cause of improvement in participants’ conditions, the proposed outcome measures will not change for the participants that remain in the baseline phase, but will be improved only for those in the intervention phase.

#### Baseline

As depicted in Fig 1, all patients will begin the baseline phase simultaneously. Each patient will undergo a baseline phase of a different time duration, starting with three weeks for the first participant and gradually increasing by one week for each participant. During the baseline phase, each participant will be assessed on the Modified Fatigue Impact Scale [53] during the first week. The neurophysiological (i.e., CMCT, resting motor threshold, MEP amplitude and latency) and clinical (i.e., gait, balance, strength, hand dexterity, cognitive functions) assessments will be repeated after each baseline week for all participants.

#### Intervention

Immediately after the end of each baseline phase, the intervention phase will begin staggered across participants and time accordingly (Fig 1). The intervention protocol consists of exercises based on in-phase bilateral movements of the upper limbs, which are adapted to different sport activities and to fitness functional exercises, organized in a circuit training considering the MS exercise recommendations [61]. Since no established protocols have been previously reported, for the needs of our study a certified fitness instructor will design these protocols adapted to different sport activities. Specifically, each session will consist of one to three sets, consisting of 10–15 repetitions of 9 different exercises targeting large muscle groups of the upper limbs (shoulder flexors, extensors, rotators, abductors and adductors, elbow flexors and extensors, hand and finger flexors and extensors). Additionally, three exercises will target large lower limbs muscle groups (hip flexors, extensors, abductors and adductors, knee and ankle flexors and extensors) to be performed in between the upper limbs exercises and to allow relaxation of the upper limbs muscles.

The specific exercises will include sports activities of basic technical skills of basketball (e.g., different types of passing, catching and throwing the ball) and volleyball (e.g., different types of passing and receiving the ball), whereas the fitness exercises will include shoulder rows, shoulder lateral raises, elbow flexions, elbow extensions, as well as using the diagonal movements form proprioceptive neuromuscular facilitation technique [62] by the use of resistance elastic bands [49], as well as exercises with the patients’ own body weight (e.g., pushups, TRX) [63]. To maintain the interest of the participants, the exercise program will be modified throughout the course of the 12-week intervention period via changing the level of difficulty. For example, we will use elastic bands with different resistance levels, the number of repetitions and sets will vary along with the specific exercise and body position (e.g., sitting or standing).

The intervention phase for each participant will consist of 12 consecutive weeks in which the proposed protocol will be performed three times per week, for 30–60 minutes each session, adapted to each participant’s fatigue and fitness level. Each participant has to complete at least 27 (75%) out of 36 sessions in order to be included in the data analysis [49]. Every intervention session will consist of a five minutes’ warm-up (i.e., whole body range of motion exercises), followed by the main sport activities and fitness exercise protocol as described above, and a cool down for five minutes (i.e., passive stretching exercises of the muscle groups which are involved in the main part).

Additionally, starting from the third intervention week, we will perform five neurophysiological and five clinical assessments (i.e., once a week), to collect five data points for every participant across the intervention phase. Moreover, each participant will also be asked to complete once the Modified Fatigue Impact Scale (see secondary measures) at the end of the intervention phase [49] (Fig 1).

#### Follow-up

As depicted in Fig 1, every participant will undergo three follow-up assessments in total, after finishing the training protocol, so to explore possible long-lasting effects. Each follow-up assessment includes both neurophysiological and clinical measures. We will perform the first follow-up assessment at the end of the fourth post-intervention week, the second one at the end of the eighth post-intervention week and the last follow-up assessment at the end of the 12^th^ post-intervention week (Fig 1).

### Primary outcome measure

Since that prolongation of CMCT is the most common neurophysiological characteristic in people with MS [64] and given the results of the study of Meng et al., (2018) [65], which indicated short term improvement of the CMCT after bilateral exercises of the upper limbs in stroke survivors, we designate the CMCT as our primary outcome variable. CMCT expresses the time taken for neural impulses to reach from motor cortex to alpha-motoneurons [64], which refers to the integrity of the white matter fibres [66]. Therefore, we will calculate bilateral CMCT using both TMS and peripheral stimulation of the median nerve (see below; Data Acquisition of Outcome Measures) to observe possible changes in the central nervous system due to possible effects of our proposed intervention protocol.

### Secondary outcome measures

The secondary outcome measures will include the resting motor threshold (states the general excitability of the neuromotor axis in the target muscle), the MEP amplitude (expresses the trans-synaptic activation of corticospinal neurons) and latency (defines the time which is needed for signal transmission from the motor cortex to the recording electrode of the target muscle) [67], and all clinical assessments. We will quantify the resting motor threshold and the MEP amplitude and latency using a single pulse TMS and two independent physiotherapists will both perform all clinical assessments to each participant.

### Data acquisition of outcome measures

We will assess the corticospinal plasticity using single pulse TMS in the neurophysiology lab of the Cyprus Institute of Neurology and Genetics. Using electromyography (EMG) signals from an upper limb muscle (see below; EMG recording), we will collect MEP, which will be used to calculate all corticospinal excitability measures. During all neurophysiological assessments, participants will be in a relaxed sitting position in a comfortable chair with feet touching the floor and both arms placed on cushioned armrests and with the head rested on a cushion. To ensure methodological consistency, we will collect all data by performing the same methodological procedures for both conditions (i.e., corticospinal excitability measures bilaterally)—one side per assessment—across participants and across all time points.

#### EMG recording

During both TMS and peripheral stimulation, surface EMG of the Abductor Pollicis Brevis (APB) muscle will be collected. We will follow a standard skin preparation [68] and surface disk electrodes placement procedures by attaching the electrodes over the end plate region of the APB [69]. Specifically, the anode electrode will be placed distally, whereas the cathode electrode proximally. A ground reference electrode will be attached on the lateral condyle of the elbow, of the corresponding upper limb. Additionally, all signals will be recorded with sampling rate of 24kHz and will be filtered with a bandwidth of 2Hz–10kHz using KeyPoint Net Software Electromyography (version 2.40; Natus Medical Incorporated G4, United States).

#### Peripheral stimulation

In addition to MEP latency, calculating the CMCT requires two peripheral derived measures, the F wave (i.e., late muscle response) and the M wave (i.e., direct muscle response) [70,71]. Therefore, we will initially deliver peripheral stimulation on the median nerve at the wrist, approximately in an 8cm distance from the cathode electrode [69], while collecting EMG from the APB [72].

#### TMS assessment

Following TMS recommended guidelines concerning safety and experimental conditions [67,73], we will assess bilateral corticospinal excitability measures. We will apply TMS single pulses [74] via figure-eight coil (C-B60; inner diameter: 35mm, outer diameter: 75mm), connected to the MagPro R20 (MagVenture User Guide, United Kingdom edition, MagVenture A/S, Denmark). The coil will be oriented tangentially over the contralateral motor area of the brain, relative to the target muscle (i.e., APB), with a posterolateral handle pointing in approximately 45 degrees angle to the sagittal plane, as a result to induce current in a posterior-anterior direction in the brain [75].

For the TMS procedures, we will first find the optimal stimulation site (i.e., hot-spot), next we will determine the resting motor threshold and then apply a bout of single pulses using suprathreshold stimulation. To determine hot-spot (i.e., the spot in which the largest response of the target muscle is elicited), we will deliver single pulses at low intensities (e.g., ~20% maximum stimulator output) and gradually increase it by 1–5% maximum stimulator output until we will reach the intensity that will elicit three consecutive MEPs with peak-to-peak amplitude greater than 50mV [76,77]. Then, we will mark the position of the coil on the skull with a water-resistant ink, to determine the resting motor threshold of the target muscle. Resting motor threshold is the minimum stimulation intensity needed to produce MEPs of the target muscle. To identify the resting motor threshold, we will employ an adaptive threshold-hunting method, the Motor Threshold Assessment Tool (MTAT 2.0) [78] (available at http://clinicalresearcher.org/software.htm). The specific method has the advantage of speed without losing accuracy when compared to the relative-frequency methods based on the Rossini–Rothwell, although both methods have similar precision [79,80]. Then, to quantify the MEP-derived measures of interest (i.e., MEP amplitude and latency), we will apply 25 suprathreshold stimuli [81] at 120% of the resting motor threshold [82].

#### Clinical assessment

We will perform all clinical assessments in the physiotherapy unit of the Cyprus Institute of Neurology and Genetics. Two independent physiotherapists will both perform all clinical assessments to each participant, with the exact same methodological procedures, in order to ensure validity of the results [54]. However, the two assessors will perform two clinical assessments to each participant prior to the beginning of the baseline phase. These two clinical assessments will not be included to the data analysis, but they will be used as a training to the participants in order to eliminate variability of the outcome measures between the different baseline durations.

*1*. *Mini Balance Evaluation Systems Test*. It measures dynamic balance, functional mobility and gait in neurological patients, including people with RRMS [83]. The specific test consists of 14 items, including four of the six segments (anticipatory postural adjustments, sensory orientation, reactive postural control and dynamic gait) from the Balance Evaluation Systems Test. The Mini Balance Evaluation Systems Test should be scored out of 28 points to include 14 items that are scored from zero to two.

*2*. *Six Spot Step Test*. It is a timed walking test that involves kicking over a number of targets placed along a 5m-path in which rely to some extent on vision and cognition [84]. The Six Spot Step Test is measured in the time domain replicating a complex range of sensorimotor functions, part of which are lower limb strength, spasticity, coordination, as well as balance. We will perform the specific test as described by Nieuwenhuis et al. (2006) [84] and record the mean time of the four runs as the final test result [85].

*3*. *Action Research Arm Test*. It is a 19-item observational measure used by physiotherapists and other health care professionals to examine upper limb performance (i.e., coordination, dexterity and functioning) [86]. Items covering the Action Research Arm Test are categorized into four subscales (grasp, grip, pinch and gross movement) and arranged in order of decreasing difficulty, with the most difficult task examined first, followed by the least difficult task. The patient is sitting comfortable in front of a stable desk performing each task and the performance is rated on a four-point scale, ranging from 0 (no movement) to 3 (movement performed normally). We will record the total score for each upper limb separately as the final test result.

*4*. *Isometric Dynamometer*. We well assess the isometric muscle force of major muscle groups with the use of the muscle controller (Kinvent Biomechanique, Montpelier, France), which is a dynamometer used in the evaluation and rehabilitation of muscle strength that provides real time biofeedback [87]. The patient lies (supine or prone) on a therapeutic bed and the physiotherapist, with the use of the muscle controller, holds against the patient’s limb as the patient exerts a maximal force. The physiotherapist counters the force (make test) or tries to break the contraction (break test) and the data will be stored using the KFORCE APP (Kinvent Biomechanique, Montpelier, France). Shoulder flexors, extensors, rotators, horizontal adductors and abductors, and elbow flexors and extensors are the major muscle groups which will be evaluated. A separate value for each muscle group will be recorded in order to be used in the data analysis.

*5*. *Symbol Digit Modalities Test*. We will employ the oral form of the test, which assesses the information processing speed [88]. During the test, the participant will be given two minutes to orally match symbols with digits as quickly as possible. The key (specifying which symbols are assigned to which numbers) will be located at the top of a computer screen. The researcher will instruct the participants that each symbol is paired with a digit. Next, the participant will be instructed to perform the test by responding orally to each symbol. For example, the symbol “O” is matched with the number “6”, so the correct response would be to say “six”. The researcher responsible for clinical assessments will record the participant’s responses directly on a computer screen. The score is obtained by subtracting the number of errors from the number of items completed in two minutes.

*6*. *Modified Fatigue Impact Scale*. It is a short questionnaire which requires the participants to describe the effects of fatigue during the past four weeks [53] (S1 Appendix). The Modified Fatigue Impact Scale consists of 21 questions which are subjectively rated from “0” (low rate) to “4” (high rate) and it is also divided into three subscales (i.e., physical, cognitive, and psychosocial). We will record the total score of the test as the final test result. The higher the score is, the greater is the impact of fatigue in individual daily life. Therefore, we will use the Modified Fatigue Impact Scale as the description of participants`attribution of functional restrictions to fatigue symptoms.

### Analysis plan

To investigate possible effects of our protocol we will follow recommended guidelines [52], in which we will perform a separate analysis for each of the outcome measures, in all experimental phases (i.e., baseline, intervention and follow up). We will perform a visual analysis first, in order to determine whether there is a functional relationship between the intervention and the outcome measures. Then, if the visual analysis will indicate a potential sizeable effect, we will perform statistical analysis to evaluate the magnitude of the intervention effect [52].

#### TMS measures analysis

Corticospinal plasticity will be determined through changes of the corticospinal excitability measures. Hence, we will quantify bilateral resting motor threshold, MEP amplitude and latency, and CMCT, because each measure can assess different plastic changes across the neuromotor axis and they can be used as a proxy of corticospinal plasticity. Resting motor threshold (% maximum stimulator output) is the lowest intensity needed to elicit a MEP from a single-pulse TMS [73], amplitude (mV) is the difference in voltage between the maximal negative to maximal positive deflection of a MEP, which is referred as peak-to-peak amplitude [73], latency (ms) is the time between the TMS onset and the MEP onset [64], while CMCT (ms) estimates the conduction time of corticospinal fibres between motor cortex and alpha-motoneurons [31].

For both upper limbs, corticospinal excitability measures (i.e., MEP amplitude and latency) will be first calculated from each MEP trace and then averaged to get a single value. These calculations will be done according to the different time points for each participant in the baseline phase, at five time points in the intervention phase and at three time points in the follow-up phase (Fig 1). In order to investigate possible changes in corticospinal excitability, we will measure resting motor threshold and calculate peak-to-peak amplitude throughout assessing MEPs [89] of the APB, while measuring of latency will indicate possible changes in CMCT. Any changes in all measures across time points, will indicate alterations in corticospinal plasticity [90]. We will evaluate resting motor threshold using MTAT 2.0 [78] (available at http://clinicalresearcher.org/software.htm) and to investigate possible changes in individual corticospinal plasticity of each participant, we will calculate bilaterally the difference between the mean values of each phase [75,91]. On the other hand, from each stimulus response during the suprathreshold stimulation (i.e., 120% of resting motor threshold) [90], we will calculate offline the MEP peak-to-peak amplitude and latency. To define CMCT (ms), we will subtract the peripheral conduction time ((F wave latency + M wave latency– 1)/2) from the MEP latency. F-wave is the response of the targeted muscle produced by antidromic activation of motoneurons following the peripheral stimulation of motor nerve fibres, whereas M wave produced by the direct muscle response [70–72]. A prolonged CMCT indicates damage of large fibres, demyelination of central motor pathways or slow summation of descending excitatory potentials in the corticospinal tract evoked by TMS [70,92]. To standardise the latencies of all motor responses derived from different stimulation protocols (i.e., MEP, F and M wave), we will use a visual inspection from stimulation onset to response onset, performed from the same investigator so to ensure reliability and reproducibility of these measures across all time points. To define possible changes in CMCT, we will evaluate bilaterally the difference between the mean values of each phase.

#### Clinical measures analysis

For each clinical measure (i.e., balance, gait, cognitive functions, bilateral hand dexterity, strength and the Modified Fatigue Impact Scale) we will calculate the values from each time point across all phases (i.e., baseline, intervention, follow-up) and then we will evaluate the average of them, so to get a single mean value for each measure and for each phase (i.e., mean baseline; mean intervention; mean follow-up). To investigate the association between the intervention protocol and clinical condition, we will calculate the differences between phases`mean values (i.e., mean baseline; mean intervention; mean follow up), reflecting to the degree of change in clinical condition following in-phase bilateral exercises.

### Visual analysis

Two independent assessors, will systematically measure each outcome measure across time, inter-assessor agreement will be calculated on at least twenty percent of the data points in each condition. The minimum acceptable inter-assessor agreement will be set to 0.8 [54].

Initially, a visual analysis will be conducted and data will be presented graphically in spaghetti plots, in order to define whether there is a functional relation between the intervention and the outcome measures [52]. During the visual analysis, six features of the research design graphed data will be examined: level, trend, stability, immediacy of the effect, overlap, and consistency. Over the within-phase examination an evaluation of level, trend and stability will be examined. Level will be reported from the mean score of each dependent variable and trend will determine whether the data points are monotonically decreased or increased. To quantify the within phase differences in level and thus to identify whether there would be substantial increase in the targeted behaviors, we will use the Percentage of data Exceeding the Median [93]. Stability will be estimated based on the percentage of data points falling within 15% of the phase median, if this is higher than 80% then we assume that this criterion is met. Additionally, over the between-phase examination an evaluation of overlapping data among baseline and intervention phases, consistency of data patterns and immediacy of effect will be performed [52]. Immediacy of the effect will be examined by comparing changes in level between the last three data points of one phase and the three first data points of the next phase. Furthermore, consistency of data patterns involves the observation of the data from all phases within the same condition, with greater consistency expressing greater causal relation. Each feature will be assessed individually and collectively across to all participants and all phases. Consequently, if the intervention protocol is the sole determinant of improvement, we expect to find indicators of improvement only at the intervention phase.

### Statistical analysis

For each of the outcome variables we will perform a visual analysis to test for any effects due to the intervention. If the visual analysis will indicate potential functional effects, we will next use the Nonoverlap of All Pairs metric in order to estimate the effect of the intervention and randomization tests will be constructed to evaluate statistical significance [52,60,92].

The null hypothesis is that there is no improvement from the proposed intervention, thus participants’ responses are independent from the condition (baseline versus intervention) under which they were observed. The alternative hypothesis is that the neurophysiological parameters and/or the clinical condition of the participants will be affected by the specific intervention, assessed separately. We will reject the null hypothesis if the p value is smaller than the Bonferroni corrected p-value based on the actual number of tests that will be performed (0.05/number of tests). All tests will be two sided. Statistical analysis will be performed using the statistical software R (https://www.r-project.org/).

## Possible threats

During the study implementation, different threats might be present which could affect internal validity of the study [54].

Attrition is one threat [54], which might have an impact on the experimental conditions in the case of less than three participants and less than three data points in each phase will present [60]. Given that, we proposed a specific methodology, which includes five participants and at least three assessments points per participant, throughout all phases (i.e., baseline, intervention, follow-up) so to avoid attrition (Fig 1). Additionally, according to our proposed protocol, participants have to complete at least 75% of the total intervention sessions, therefore this will not affect the implementation of our study in case of an absent during the intervention phase.

History is another possible threat [54]. Because we might have a limited ability to explore what other events probably will influence the outcome measures, we will ask from each participant to have a written calendar of their daily routine (e.g., any other physical activity, occupational and pharmaceutical changes) throughout the study duration. Also, by using the specific proposed study design (i.e., single-case multiple baseline design) we eliminate the present of this thread, because we have the advantage to monitor and examine individual behaviour through the repetitively data collection during baseline and intervention phases. Moreover, to ensure that participants will not make other outcome-related changes in their daily life, they will be advised prior to the study implementation to continue their usual prescribed medication throughout the study duration. However, if a participant will make any changes to their usually prescribed medication upon physician recommendation, the exact period of the particular change will be noted in order to relate any possible change to the outcome measures.

## Supporting information

S1 Appendix Modified fatigue impact scale. (PDF)

S2 Appendix SPIRIT checklist. (PDF)

S3 Appendix Study protocol. (PDF)
