# Supplementary material for: Investigation of in-phase bilateral exercise effects on corticospinal plasticity in relapsing remitting multiple sclerosis: A registered report single-case concurrent multiple baseline design across five subjects
Source: PLoS One. 2023 Mar 2;18(3):e0272114. doi: 10.1371/journal.pone.0272114 (PMC9980831; doi:10.1371/journal.pone.0272114)
Supplement: S1 Appendix — (PDF) [file pone.0272114.s001.pdf]

Appendix 1

Modified Fatigue Impact Scale (MFIS)

ID: __________ Date: __________

Below is a list of statements that describe the effects of fatigue during the past four weeks. Please read each statement carefully and circle the number which indicates how often fatigue has affected you in this way. You have to answer every question, but if you are not sure about an answer, select the one that comes closest to your description.

Because of my fatigue during the past 4 weeks

No.	Statement	Never	Rarely	Sometimes	Often	Almost Always
1	I have been less alert	0	1	2	3	4
2	I have had difficulty paying attention for long periods of time.	0	1	2	3	4
3	I have been unable to think clearly.	0	1	2	3	4
4	I have been clumsy and uncoordinated.	0	1	2	3	4
5	I have been forgetful.	0	1	2	3	4
6	I have had to pace myself in my physical activities.	0	1	2	3	4
7	I have been less motivated to do anything that requires physical effort.	0	1	2	3	4
8	I have been less motivated to participate in social activities.	0	1	2	3	4
9	I have been limited in my ability to do things away from home.	0	1	2	3	4
10	I have trouble maintaining physical effort for long periods.	0	1	2	3	4
11	I have had difficulty making decisions.	0	1	2	3	4
12	I have been less motivated to do anything that requires thinking.	0	1	2	3	4
13	My muscles have felt weak.	0	1	2	3	4
14	I have been physically uncomfortable.	0	1	2	3	4
15	I have had trouble finishing tasks that require thinking.	0	1	2	3	4
16	I have had difficulty organizing my thoughts when doing things at home or at work.	0	1	2	3	4

17	I have been less able to complete tasks that require physical effort.	0	1	2	3	4
18	My thinking has been slowed down.	0	1	2	3	4
19	I have had trouble concentrating.	0	1	2	3	4
20	I have limited my physical activities.	0	1	2	3	4
21	I have needed to rest more often or for longer periods.	0	1	2	3	4

Instructions for Scoring

All items can be aggregated into three subscales (i.e., physical, cognitive, and psychosocial), as well as into a total score. The higher the score is, the greater is the impact of fatigue on a participant`s activities.

1. Physical Subscale

This scale can range from 0 to 36. It is calculated by adding the scores of the following items: 4+6+7+10+13+14+17+20+21 = ...

2. Cognitive Subscale

This scale can range from 0 to 40. It is calculated by adding the scores of the following items: 1+2+3+5+11+12+15+16+18+19 = ...

3. Psychosocial Subscale

This scale can range from 0 to 8. It is calculated by adding the scores of the following items: 8+9 = ...

Total Score

The total score can range from 0 to 84. It is calculated by adding the scores of the physical, cognitive and psychosocial subscales.

Total score = ...
