# Supplementary material for: Investigation of in-phase bilateral exercise effects on corticospinal plasticity in relapsing remitting multiple sclerosis: A registered report single-case concurrent multiple baseline design across five subjects
Source: PLoS One. 2023 Mar 2;18(3):e0272114. doi: 10.1371/journal.pone.0272114 (PMC9980831; doi:10.1371/journal.pone.0272114)
Supplement: S3 Appendix — (PDF) [file pone.0272114.s003.pdf]

Appendix 3_Study protocol EEBK.ΕΠ.2022.32

Protocol of study "The investigation of the effects of different types of exercise in neuroplasticity and in clinical condition, in people with Multiple Sclerosis".

Multiple sclerosis (MS) is the most common inflammatory, demyelinating and neurodegenerative disease of the central nervous system (CNS) (1). The prevalence of MS during the last two decades, has significantly increased all over the world (2,3), with predominance among young people (4), The low mean age of diagnosis (i.e., 32 years old), along with an average of seven years' shorter life expectancy (i.e., 74.7 years) compared to the general population (5–7), highlights the need for a lengthy support, resulting in increased financial and social burden (4). Furthermore, both MS patients and their caregivers, are facing several psychological and social difficulties due to social isolation, poorer quality of life (QoL) (8,9).

Relapsing-remitting (RR) MS is a type of MS (10) which is characterized by periods of relapses followed by partial or complete recovery (1). However, a great amount of patients diagnosed with RRMS transit to secondary-progressive MS (SPMS) within 10 years, whereas some others transit within 25 years (1). Individual clinical condition varies between pwMS and symptoms are diverse, including motor impairments, cognitive dysfunctions, vision deficits, depression and fatigue (11–13), causing neurological disability which results in reduction of QoL (14,15). Physical disability and especially motor symptoms in pwMS, are associated with changes in the corticospinal tract (16–21), which is the major neuronal pathway providing voluntary motor function in human (22). Nevertheless, motor symptoms in pwMS can be managed with improvement of neuroplasticity (23–26), which is defined as the ability of the CNS to continuously adapt to changes in the environment (27). Moreover, neuroplasticity has an essential role in clinical condition of pwMS, due to the fact that greater values of neuroplasticity in the stable stage of the disease, can be a strong predictor for a better clinical recovery in the post-relapse stage (28,29). These neuroplastic effects in pwMS, are exerted throughout the propagation of neural signals in the corticospinal system (30), which are associated with the corticospinal tract integrity. To our knowledge, corticospinal plasticity can be probed via Non-Invasive Brain Stimulation techniques, specifically via Transcranial Magnetic Stimulation (TMS) (27). Therefore, corticospinal plasticity can be examined throughout a number of TMS-based neurophysiological measures that exhibit abnormalities in corticospinal excitability, including abnormal motor threshold (MT) and altered central motor conduction time (CMCT) (30).

As far as we know, corticospinal plasticity is exercise-depended activity (31,32) affected by various factors (33,34), including the different physiotherapeutic approaches (24,35), aerobic exercise (26,36–38) resistance training (26,38) as well as interlimb coordination (39,40). Additionally, several studies (41–44) using healthy participants and chronic stroke survivors (41,42,44,45) reported that bilateral movement instead of unilateral, leads to the greatest increase in corticospinal plasticity due to suppression of the cortical inhibition (45,46) and due to simultaneous activation of homologous representations of the motor cortices (47,48). Still, considering the importance of corticospinal plasticity in the clinical condition of pwMS and due to the fact that cortical lesions can be detected unexpected in both brain hemispheres (49), there is a gap regarding the investigation of the effects of the bilateral movements in corticospinal plasticity and the impact in motor and cognitive functions, in pwMS.

Therefore, the aim of this study is to investigate bilateral corticospinal changes and possible effects in motor and cognitive functions in pwMS, during a 12-weeks different intervention programs based on bilateral versus unilateral movements, adapted to sports activities exercises and fitness functional exercises. Consequently, we assume that possible effects from our study will promote a novel approach in the field of neurorehabilitation and will introduce an innovative type of exercise during the stable stage of the disease.

This research study follows a concurrent multiple baseline design across subjects, which is designed according to the 'What Works Clearinghouse' criteria for single case studies (50). All participants will begin the study with the baseline phase at the same time while the intervention phase is introduced staggered across patients and time. The intervention is introduced systematically in one patient while baseline data collection continues in the others without any intervention. The cause-effect inference can be clearly verified by the staggered duration through separate baseline phases (51). Subsequently, if the intervention (independent variables) will be the only element of improvement in participant`s conditions, the proposed outcome measures (depended variables) will not change for the participants that remain in the baseline phase, but will be improved only for those in the intervention phase. The efficacy of our proposed treatment protocol will be assessed by a single-case experimental design study. Single case methodology refers to the intensive study of one or more participants and recently was ranked as Level 1 evidence for treatment decision purposes (52). Accordingly, it is suggested that properly designed and executed single case studies, have the capacity to demonstrate cause-effect relationships between the independent and the dependent variables, with relatively small sample (50).

The study will take place at the physiotherapy unit of the Cyprus Institute of Neurology and Genetics (CING). All experimenters in the proposed study are health care professionals. All data storage and statistical analysis will take place at the facilities of the Department of Rehabilitation Sciences of Cyprus University of Technology (CUT) and at the physiotherapy unit of the CING. All of the measures are to be administered by the clinical research team member (senior physiotherapist) under the supervision of the project coordinator. All data and personal information collected from the study will be stored safely at the premises of CUT and CING and not shared between researchers. Five years after data collection the measurements will be safely destroyed.

Participants and methods

We will recruit 30 pwMS in total, over the course of five years. The study includes two groups of pwMS, the first one contains 15 people with RRMS and the second one 15 people with SPMS. Each individual from this group will be allocated respectively in another three subgroups of 5 participants according to the type of movement (i.e., subgroup 1: in-phase bilateral sport activities and fitness functional exercises, subgroup 2: anti-phase bilateral sport activities and fitness functional exercises, subgroup 3: unilateral sport activities and fitness functional exercises). Firstly, we will recruit the group of people with RRMS who will participate in the three subgroups with same order as mentioned above. After the first group will finish, we will recruit the group of people with SPMS who will also participate in the three subgroups with same order as mentioned above. The number of participants in each subgroup is justified based on the 'What Works Clearinghouse' criteria for single case studies (50).

Fifteen people with RRMS and Expanded Disability Status Scale (EDSS) (53) score between 3-5 and 15 people with SPMS and EDSS between 3-6 will be

included in the study. All participants will be recruited from the data base of the CING and especially through a referral of the neurologist from the CING, who is the clinical coordinator of the Neuroepidemiological Department and she will evaluate also the EDSS for the needs of this study. The inclusion criteria include 1) people with RRMS with EDSS score between 3–5, 2) people with SPMS with EDSS score between 3-6, 3) aged between 30 and 70 years old, 4) no relapse within 30 days and 5) Mini Mental State of Examination (MMS) score between 24-30 (no cognitive impairment). The exclusion criteria include 1) metal implants, 2) history of any disease affecting the central nervous system other than MS, 3) history of cardiovascular disease, 4) mental disorders, 5) severe orthopedic disorders, 6) pregnancy, 7) visual deficit, 8) hearing impairments, 9) epileptic seizures and 10) spasticity level on upper or lower limbs more than 1+ (slight increase in muscle tone) according to Modified Ashworth Scale (54). Additionally, participants will be advised to continue their usual prescribed medication throughout the study duration and they will be advised to continue their usual routine, avoiding to receive any other exercise program during the study.

All participants will be required to provide consent, after being informed about the process of filing complaints. The experimenter will assign a participant ID number (e.g., Participant 101, 102, etc.). From this point on, the name of each participant will not be used again and only the participant ID number will be used for statistical analyses or other reasons. Subjects, patients and carer/family will be provided with the contact details of the Director of Research and International Relations of the CUT, an independent authority that is unrelated to the study, in the case they wish to make anonymous or branded comments or complaints about this research project. Moreover, subjects will be informed in the consent form that they can be informed about the results of the study relevant to them, following completion of the study. Consent will be perceived by the participants as they are adults without cognitive deficits (see inclusion criteria) and also they have the ability to understand, think, express and write. The participants who will be included in the study will be given a detailed description and explanation of all procedures, analysis of the purpose of the study as well as the benefits and rights that each will have through his/her participation. Finally, all the gathered data will be stored at the principal investigator's office and only the investigators of the study will have access to participants' name and data. Five years after data collection the measurements will be safely destroyed.

The intervention protocols contain bilateral and unilateral movements of the upper limbs which are adapted to exercises based on different sport activities and fitness functional exercises. Regarding the exercises which will be included in the sport activities, the participants will be practiced in basketball, volleyball, boxing and swimming. Due to the fact that no set protocols have been reported regarding the previous mentioned activities, for the needs of this study certified coaches will design adapted protocols related to each sport activity, based on previous literature. Regarding the boxing, an adapted protocol will be designed based on the study of Sangarapillai et al., (55). Concerning the basketball, an adapted protocol related to the basic technical skills will be designed (e.g. different types of dribbling, passing and catching the ball, shooting, relay racing etc). Similarly, an adapted protocol related to the volleyball will be designed based on the basic technical skills (e.g. different types of passing, serving, receiving the ball). Finally, an adapted protocol will also be designed regarding the basic swimming skills from the three main swimming strokes (i.e. crawl, breaststroke, backstroke). On the other hand, the exercises which will be performed regarding the fitness part will be based on a circuit functional training, including exercises with the use of light resistance bands (e.g. towards movement directions of the shoulder, elbow and wrists joints) with the use of their own body weight (e.g.

pushups, TRX) (56). The intervention phase for each participant consists of 12 consecutive weeks in which the proposed protocol will be performed three times per week, for 30-60 minutes each session, in respect of individual levels of fatigue. However, the duration and the intensity of the exercises will be designed considering previews literature (37,57) and recommendations (58). Additionally, for each participant we will perform several neurophysiological and clinical assessments during baseline, intervention and follow up phases. The neurophysiological assessments include evaluation of corticospinal plasticity via TMS (27,30). Corticospinal plasticity characterized via certain TMS-specific neurophysiological measures including resting MT (rMT), motor-evoked potential (MEP) amplitudes and the CMCT (30). Moreover, clinical assessments will be gathered regarding motor skills (i.e., walking, balance, strength, upper limb dexterity) and cognitive functions (i.e., IPS), as well as a subjective questionnaire will be provided to the participants related to fatigue. Age, gender and disease duration since diagnosis will be collected as descriptive measures.

The benefits that all participants will gain are the systematic evaluation of their clinical condition for a long period of time, as well as their participation for a long time in an exercise program which will keep them physically active.

The expected results of this research are:
1) Improvement of motor functions (gait, balance, strength, upper extremity functionality).
2) Improvement cognitive functions (information processing speed).
3) Possibility to increase the plasticity index in both cerebral hemispheres, which means that each patient will have a much better prognosis in a possible relapse, due to the nature of the disease, as well as a qualitative recovery in a such situation.
4) Potential reduction of the CMCT in which there will be an improvement in the control of several motor functions, mainly of the upper limbs.
5) All participants will also benefit from a number of clinical evaluations and exercise sessions, which are much more in quantity than what they are funded throughout the General Health System.

Study design

Each subgroup includes five participants and each one constitutes a separate research study that follows a concurrent multiple baseline design across subjects, designed according to the 'What Works Clearinghouse' criteria for single case studies (50). During the experimental procedure, all participants will begin the study with the baseline phase at the same time while the intervention phase is introduced staggered across patients and time (see Figure 1). The intervention will be introduced systematically in one patient while baseline data collection continues in the others without any intervention. The cause-effect inference can be clearly verified by the staggered duration through separate baseline phases (51). Subsequently, if the intervention is the sole cause of improvement in participants' conditions, the proposed outcome measures will not change for the participants that remain in the baseline phase but will be improved only for those in the intervention phase.

Data Acquisition of Outcome Measures

Primary Outcome Measures

We will assess the corticospinal plasticity using single pulse TMS in the neurophysiology lab of the Cyprus Institute of Neurology and Genetics, in all phases (see Figure 1). Using electromyography (EMG) signals, we will analyze bilateral cortical excitability and bilateral CMCT

to determine corticospinal plasticity and therefore to test the primary hypothesis. The rMT and the MEP amplitude of Abductor Pollicis Brevis muscle will define cortical excitability, while we will use the MEP latency to calculate the CMCT. During all neurophysiological assessments, participants will be in a relaxed sitting position in a comfortable chair with feet touching the floor and both arms will be placed on cushioned armrests and with the head rested on a cushion. To ensure methodological consistency, we will collect all data by performing the same methodological procedures for both conditions (i.e., cortical excitability and CMCT) bilaterally (one side per assessment), across participants and across all time points.

TMS assessment

Following TMS recommended guidelines concerning safety and experimental conditions (59,60), we will assess bilateral cortical excitability and bilateral CMCT. We will apply TMS single pulses (61) via figure-eight coil (C-B60; inner diameter: 35mm, outer diameter: 75mm), connected to the MagPro R20 (MagVenture User Guide, United Kingdom edition, MagVenture A/S, Denmark). For the TMS procedures, we will first find the optimal stimulation site (i.e., hot-spot), next we will determine the rMT, and then apply a bout of single pulses using suprathreshold stimulation. To determine hot-spot (i.e., the spot in which the largest response of the target muscle is elicited), we will deliver single pulses at low intensities (e.g., ~20% maximum stimulator output) and gradually increase it by 5%, maximum stimulator output until we will reach the intensity that will elicit three consecutive MEPs with peak-to-peak amplitude greater than 50mV (62,63). Then, we will determine the rMT of the target muscle which is the minimum stimulation intensity needed to produce MEPs of the target muscle and it is defined by the hot-spot. To identify the rMT of the Abductor Pollicis Brevis muscle, we will employ an adaptive threshold-hunting method, the Motor Threshold Assessment Tool (MTAT 2.0) (64) (available at http://clinicalresearcher.org/software.htm). The specific method has the advantage of speed without losing accuracy when compared to the relative-frequency methods based on the Rossini–Rothwell, although both methods have similar precision (65). Then, to quantify the MEP-derived measures of interest (i.e., MEP amplitude, latency), we will apply 25 suprathreshold stimuli (66) at 120% of the rMT (67).

Secondary outcome measures

All clinical assessments will be performed in all phases (see Figure 1) in the physiotherapy unit of the CING and reliability of the measures will be assessed by having two trained physiotherapists, blinded to the research study, independently record the measures of each outcome.

Mini Balance Evaluation Systems Test (Mini-BESTest).

The Mini-BESTest (68) it is a shorter version of the Balance Evaluation Systems Test (BESTest) (69). This test measures dynamic balance, functional mobility, and gait in neurologic patients, including pwMS. The Mini-BESTest consists of 14 items, including four of the six segments (anticipatory postural adjustments, sensory orientation, reactive postural control and dynamic gait) from the BESTest. The Mini-BESTest should be scored out of 28 points to include 14 items that are scored from zero to two.

Six Spot Step Test (SSST)

The SSST (70) is a timed walking test that involves kicking over a number of targets placed along a 5m-path in which rely to some extent on vision and cognition. The SSST is measured in the time domain, replicates a complex range of sensori-motor functions, part of which are lower limb strength, spasticity, coordination, as well as balance. We will perform the specific test as it is described by Nieuwenhuis et al (2006) (70) and we will record the mean time of the four runs as the final test result (71).

Action Research Arm Test (ARAT)

The ARAT (72) is a 19-item observational measure used by physiotherapists and other health care professionals to examine upper extremity performance i.e. coordination, dexterity and functioning. Items covering the ARAT are categorized into four subscales (grasp, grip, pinch and gross movement) and arranged in order of decreasing difficulty, with the most difficult task examined first, followed by the least difficult task. The patient is sitting comfortable in front of a stable desk performing each task and the performance is rated on a four-point scale, ranging from 0 (no movement) to 3 (movement performed normally). We will record the total score for each upper limb separately as the final test result.

Isometric Dynamometer

The isometric muscle force of the major muscle groups will be assessed with the use of the muscle controller (Kinvent Biomechanique, Montpelier, France), which is a dynamometer used in the evaluation and rehabilitation of muscle strength that provides real time biofeedback. The patient lies (supine or prone) on a therapeutic bed and the physiotherapist, with the use of the muscle controller, holds against the patient's limb as the patient exerts a maximal force. The physiotherapist counters the force (make test), or tries to break the contraction (break test) and the data will be collected and stored using the KFORCE APP (Kinvent Biomechanique, Montpelier, France). Shoulder flexors, extensors, rotators, horizontal adductors and abductors, adductors and abductors, elbow flexors and extensors are the major muscle groups which will be evaluated (73).). Shoulder flexors, extensors, rotators, horizontal adductors and abductors adductors and abductors, elbow flexors and extensors are the major muscle groups which will be evaluated. A separate value for each muscle group will be recorded in order to be used in the visual and statistical analysis.

Symbol Digit Modalities Test- Oral form (SDMT)

The SDMT - oral form (74) assesses the IPS. During the test, the participant will be given two minutes to orally match symbols with digits as quickly as possible. The key (specifying which symbols are assigned to which numbers) will be located at the top of a computer screen. The physiotherapist who is responsible for all clinical assessments will record the participant's responses directly on the computer screen. The physiotherapist instructs the participants that each symbol is paired with a number and they are arranged across the row. Afterword, the participant is instructed how to response orally in the row without the numbers. For example, the symbol “O”, it is matched with the number 6, so the participant would say “6”; this next symbol “X”, is paired with the number 8, so the participant would say “8”. The participant will have the possibility to correct a mistake and continue, alongside the physiotherapist should strike through the items on which the participant makes errors. The score is obtained by subtracting the number of errors from the number of items completed in two minutes.

Questionnaire

The questionnaire which will be used is published in English, but all questions will be orally translated to Greek from the physiotherapists who will performed all clinical assessments and every response will be recorded at the participants' presence.

Modified Fatigue Impact Scale (MFIS)

It is a short questionnaire which requires the participants to describe the effects of fatigue during the past four weeks (75). The MFIS consists of 21 questions which are subjectively rated from "0" (low rate) to "4" (high rate) and it is also divided into three subscales (i.e., physical, cognitive, and psychosocial). The higher the score is, the greater is the impact of fatigue in individual daily life. Although, to describe fatigue level the Fatigue Severity Scale is more commonly used, we decided not use it because it describes severe fatigue. Therefore, we will use the Modified Fatigue Impact Scale as the description of participants` attribution of functional restrictions to fatigue symptoms.

The intervention protocol and all clinical assessments will be performed in the physiotherapy unit of the CING and reliability of the measures will be assessed by having two trained physiotherapists, blinded to the research study, independently record the measures of each outcome. Also, neurophysiological assessments will be executed and recorded from the experimenters who are trained in the use of the TMS equipment regarding the needs of the proposed study, in the neurophysiology lab of the CING, in separate days from those of the clinical assessments. During the follow-up phase both clinical and neurophysiological assessments will be performed at the same day for each participant. To note, each participant has to complete at least 27 (75%) out of 36 sessions in order to be included in the final data analysis (76). All of the measures are to be administered by the clinical research team member (senior physiotherapist) under the supervision of the project coordinator. All data and personal information collected from the study will be stored safely without being shared between researchers at the premises of CUT and CING. Five years after data collection the measurements and records will be safely destroyed.

Analysis plan

To investigate possible effects of our protocol we will follow recommended guidelines (77), in which we will perform a separate analysis for each of the outcome measures, in all experimental phases (i.e., baseline, intervention and follow up). We will perform a visual analyses first, in order to determine whether there is a functional relationship between the intervention and the outcome measures, and secondly, we will perform a quantitative analysis methods to evaluate the magnitude of the intervention effect, provided there is evidence from the visual analyses (77). We will perform all neurophysiological and clinical assessments to each participant according to the number of data points during each phase (i.e., baseline, intervention, follow up) (see Figure 1).

TMS measures analysis

Corticospinal plasticity will be determined through changes of the corticospinal excitability and the central motor conduction time. Hence, we will quantify bilateral rMT, MEP amplitude and latency, because each measure can assess different plastic changes across the neuromotor axis and they can be used as a proxy of corticospinal plasticity. Resting motor threshold (% maximum stimulator output) states the general excitability of the neuromotor axis in the target muscle,

amplitude (mV) expresses transynaptic activation of corticospinal neurons (78), while latency (ms) is the time between the TMS onset and the MEP onset, which refers to the integrity of the white matter fibers (79).

For both upper limbs, all neurophysiological measures (i.e., rMT, MEP amplitude, latency) will be first calculated from each MEP trace and then averaged to get a single value. These calculations will be done according to the different time points for each participant in the baseline phase, at five time points in the intervention phase and at three time points in the follow-up phase (see Figure 1). In order to investigate possible changes in cortical excitability, we will measure rMT and peak-to-peak amplitude throughout assessing MEPs (80) of the Abductor Pollicis Brevis muscle, while measuring of latency will indicate possible changes in CMCT. Any changes in all measures across time points, will indicate alterations in corticospinal plasticity (81). We will evaluate resting motor threshold using MTAT 2.0 (64) (available at http://clinicalresearcher.org/software.htm) and to investigate possible changes in individual corticospinal plasticity of each participant, we will calculate bilaterally the difference between the mean values of each phase (82,83). On the other hand, from each stimulus response during the suprathreshold stimulation (i.e., 120% of rMT) (81), we will calculate offline the MEP peak-to-peak amplitude and latency. To define CMCT (ms), we will subtract the peripheral conduction time ((F wave latency + M wave latency – 1)/2) from the CMCT (MEP latency). The F wave is the muscle response elicited by activation of the alpha motor neuron, while the M wave is the direct response of the muscle (84). A prolonged CMCT indicates damage of large fibres, demyelination of central motor pathways or slow summation of descending excitatory potentials in the corticospinal tract evoked by TMS (84,85). To standardise all latencies (i.e., MEP, F and M wave), we will use a visual inspection from stimulation onset to response onset, performed from the same investigator so to ensure reliability of the study across all time points. To define possible changes in CMCT, we will evaluate bilaterally the difference between the mean values of each phase.

Clinical measures analysis

For each clinical measure (i.e., balance, gait, cognitive function, bilateral hand dexterity and strength) and for the Modified Impact Scale we will calculate the values from each time point across all phases (i.e., baseline, intervention, follow-up) and then we will evaluate the average of them, so to get a single mean value for each measure and for each phase (mean baseline; mean intervention mean follow-up). To investigate the association between the intervention protocol and clinical condition, we will calculate the differences between phases` mean values (i.e., mean baseline; mean intervention; mean follow up), reflecting to the degree of change in clinical condition following in-phase bilateral exercises.

Visual analysis

Two assessors will systematically measure each outcome measure across time, inter-assessor agreement will be calculated on at least twenty percent of the data points in each condition. The minimum acceptable inter-assessor agreement will be set to 0.8 (50). Initially, a visual analysis will be conducted and presented graphically in a spaghetti plot, in order to define whether there is a functional relation between the intervention and the outcome measures (77). During the visual analysis, six features of the research design graphed data will be examined: level, trend, stability, immediacy of the effect, overlap, and consistency. Over the within-phase examination an evaluation of level, trend and stability will be examined. Level will be reported from the mean score of each dependent variable and trend will determine whether the data points

are monotonically decreased or increased. Stability will be estimated based on the percentage of data points falling within 15% of the phase median, if this is higher than 80% then we assume that this criterion is met. Additionally, over the between-phase examination an evaluation of overlapping data among baseline and intervention phases, consistency of data patterns and immediacy of effect will be performed (77). The Percentage of Non-overlapping Data index will be used to quantify the proportion of data points in the intervention phase that do not overlap with the baseline phase (86) and the test statistic will be calculated using the Improvement Rate Difference as an effect size index. Immediacy of the effect will be examined by comparing changes in level between the last three data points of one phase and the three first data points of the next phase. Furthermore, consistency of data patterns involves the observation of the data from all phases within the same condition, with greater consistency expressing greater causal relation. Each feature will be assessed individually and collectively across to all participants and to all phases. Consequently, if the intervention protocol is the sole determinant of improvement, we expect to find indicators of improvement only at the intervention phase.

Statistical analysis

For each of the outcome variables that the visual analysis indicates a potential functional effect, a quantitative analysis will be performed to estimate the effect size. This analysis will be performed for each variable at which a significant trend is shown as described in the previous section. In order to estimate the individual-level effect sizes, we will use three different methods, as suggested by 'What Works Clearinghouse' (50), the standardized mean difference (Cohen's d), the standardized mean difference with correction for small sample sizes (Hedges' g) and piecewise regression analysis which does not only reflect the immediate intervention effect, but also the intervention effect across time. Multilevel modelling, which is recommended by the 'What Works Clearinghouse' and the single case educational design, specific mean difference index will be used to estimate the magnitude of the effect across cases and compared to the effect obtained by the single level estimates (77). For all the different neurophysiological parameters and clinical conditions, evaluated separately, the null hypothesis is that "there is no improvement from the proposed intervention", thus participants' responses are independent from the condition (baseline versus intervention) under which they were observed. The alternative hypothesis is that "the neurophysiological parameters and /or the clinical condition of the participants will be affected by the specific intervention", again assessed separately. We will reject the null hypothesis if the p value is smaller than 0.05. All tests will be two sided. Statistical analyses will be performed using the statistical software R (https://www.r-project.org/).

Treatment Fidelity

Treatment fidelity will be monitored during the study to ensure that the exercise program and the outcome variables assessments will be executed as intended. This will allow as to truly test the effectiveness of our proposed protocol and hence, the danger to commit a Type 1 or Type 2 error will be reduced (87). A checklist will be developed to ensure that all the key elements of our study are implemented as planned. The assessors and the providers of the intervention protocol will undergo preparation to confirm their ability acquirement. During the intervention sessions, a supervisor will observe the procedure to confirm the constant and accurately administration of the intervention. During the different phases the patients will be closely monitored to identify possible ingredients that may influence the effectiveness of the intervention (e.g., changes in the pharmacological treatment, relapse, participation in other training programs, changes in their daily routine).

References

1. Lublin FD, Reingold SC. Defining the clinical course of multiple sclerosis: Results of an international survey. Neurology. 1996;46(4):907–11.
2. Moghaddam VK, Dickerson AS, Bazrafshan E, Seyedhasani SN. Socioeconomic determinants of global distribution of multiple sclerosis : an ecological investigation based on Global Burden of Disease data. 2021;1–11.
3. Walton C, King R, Rechtman L, Kaye W, Leray E, Marrie RA, et al. Rising prevalence of multiple sclerosis worldwide : Insights from the Atlas of MS , third edition. :1–6.
4. Dobson R, Giovannoni G. Multiple sclerosis – a review. Eur J Neurol. 2019;26(1):27–40.
5. Lunde HMB, Assmus J, Myhr KM, Bø L, Grytten N. Survival and cause of death in multiple sclerosis: A 60-year longitudinal population study. J Neurol Neurosurg Psychiatry. 2017;88(8):621–5.
6. Scalfari A, Knappertz V, Cutter G, Goodin DS, Ashton R, Ebers GC. Mortality in patients with multiple sclerosis. Neurology. 2013;81(2):184–92.
7. Kingwell E, Zhu F, Evans C, Duggan T, Oger J, Tremlett H. Causes that Contribute to the Excess Mortality Risk in Multiple Sclerosis: A Population-Based Study. Neuroepidemiology. 2020;54(2):131–9.
8. Maguire R, Maguire P. Caregiver Burden in Multiple Sclerosis: Recent Trends and Future Directions. Curr Neurol Neurosci Rep. 2020;20(7).
9. Kouzoupis AB, Paparrigopoulos T, Soldatos M, Papadimitriou GN. The family of the multiple sclerosis patient: A psychosocial perspective. Int Rev Psychiatry. 2010;22(1):83–9.
10. Lublin FD, Coetzee T, Cohen JA, Marrie RA, Thompson AJ. The 2013 clinical course descriptors for multiple sclerosis: A clarification. Neurology. 2020;94(24):1088–92.
11. Kister I, Bacon TE, Chamot E, Salter AR, Cutter GR, Kalina JT, et al. Multiple Sclerosis Symptoms. 2013;(June 2011):146–57.
12. Norbye AD, Midgard R, Thrane G. Spasticity, gait, and balance in patients with multiple sclerosis: A cross-sectional study. Physiother Res Int. 2020;25(1):1–9.
13. Benedict RHB, Amato MP, Deluca J, Geurts JJG. Cognitive impairment in multiple sclerosis : clinical management , MRI , and therapeutic avenues. Lancet Neurol [Internet]. 2020;19(10):860–71. Available from: http://dx.doi.org/10.1016/S1474-4422(20)30277-5
14. Frndak SE, Kordovski VM, Cookfair D, Rodgers JD, Weinstock-Guttman B, Benedict RHB. Disclosure of disease status among employed multiple sclerosis patients: Association with negative work events and accommodations. Mult Scler J. 2015;21(2):225–34.
15. Strober L, Chiaravalloti N, Moore N, Deluca J. Unemployment in multiple sclerosis (MS): Utility of the MS Functional Composite and cognitive testing. Mult Scler. 2014;20(1):112–5.
16. Kerbrat A, Gros C, Badji A, Bannier E, Galassi F, Labauge P, et al. Multiple sclerosis lesions in motor tracts from brain to cervical cord : spatial distribution and correlation with disability. 2020;2089–105.

17. Zackowski KM, Chodkowski BA, Calabresi PA. Corticospinal Tract Abnormalities Are Associated with Weakness in Multiple Sclerosis. 2008;333–9.

18. Tovar-moll F, Evangelou IE, Chiu AW, Auh S, Chen C, Ehrmantraut M, et al. Diffuse and Focal Corticospinal Tract Disease and Its Impact on Patient Disability in Multiple Sclerosis. 2014;14–6.

19. Fritz NE, Keller J, Calabresi PA, Zackowski KM. NeuroImage : Clinical Quantitative measures of walking and strength provide insight into brain corticospinal tract pathology in multiple sclerosis. NeuroImage Clin [Internet]. 2017;14:490–8. Available from: http://dx.doi.org/10.1016/j.nicl.2017.02.006

20. Pawlitzki M, Neumann J, Heidel J, Stadler E, Sweeney-reed C, Sailer M. Loss of corticospinal tract integrity in early MS disease stages. 2017;0.

21. Shanahan CJ, Walt A Van Der, Boonstra FMC, Glarin R, Kilpatrick TJ, Geurts JJG, et al. B RAIN AIN COMMUNICATIONS Axonal loss in major sensorimotor tracts is associated with impaired motor performance in minimally disabled multiple sclerosis patients. 2021;

22. Lemon RN. Descending pathways in motor control. Annu Rev Neurosci. 2008;31(Cm):195–218.

23. Lipp I, Tomassini V. Neuroplasticity and motor rehabilitation in multiple sclerosis. 2015;6(March):1–3.

24. Flachenecker P. Clinical implications of neuroplasticity - the role of rehabilitation in multiple sclerosis. Front Neurol. 2015;6(MAR):1–4.

25. Tomassini V, Matthews PM, Thompson AJ, Fuglø D, Geurts JJ, Johansen-berg H, et al. Neuroplasticity and functional recovery in multiple sclerosis. Nat Publ Gr [Internet]. 2012;8(11):635–46. Available from: http://dx.doi.org/10.1038/nrneurol.2012.179

26. Tavazzi E, Cazzoli M, Pirastru A, Blasi V, Rovaris M, Bergsland N, et al. Neuroplasticity and Motor Rehabilitation in Multiple Sclerosis : A Systematic Review on MRI Markers of Functional and Structural Changes. 2021;15(October).

27. Pascual-Leone a, Tarazona F, Keenan J, Tormos JM, Hamilton R, Catala MD. Transcranial magnetic stimulation and neuroplasticity. Neuropsychologia [Internet]. 1999;37(2):207–17. Available from: http://www.ncbi.nlm.nih.gov/pubmed/10080378

28. Mori F, Kusayanagi H, Nicoletti CG, Weiss S, Marciani MG, Centonze D. Cortical plasticity predicts recovery from relapse in multiple sclerosis. Mult Scler J. 2014;20(4):451–7.

29. Mori F, Rossi S, Piccinin S, Motta C, Mango D, Kusayanagi H, et al. Synaptic plasticity and PDGF signaling defects underlie clinical progression in multiple sclerosis. J Neurosci. 2013;33(49):19112–9.

30. Neva JL, Lakhani B, Brown KE, Wadden KP, Mang CS, Ledwell NHM, et al. Multiple measures of corticospinal excitability are associated with clinical features of multiple sclerosis. Behav Brain Res [Internet]. 2016;297:187–95. Available from: http://dx.doi.org/10.1016/j.bbr.2015.10.015

31. Zeller D, Classen J. Plasticity of the motor system in multiple sclerosis. Neuroscience [Internet]. 2014;283(June):222–30. Available from: http://dx.doi.org/10.1016/j.neuroscience.2014.05.043

32. Zentgraf K, Helm F. Brain Changes in Response to Exercise - Methodologies for Identifying the Physiological Effects of Physical Exercise. 2020;II:815–31.

33. Moucha R, Ã MPK. Cortical plasticity and rehabilitation. 2006;

34. Prosperini L, Filippo M Di. Beyond clinical changes: Rehabilitation-induced neuroplasticity in MS. Mult Scler J. 2019;25(10):1348–62.

35. Marta Niwald EM. Novel Physiotherapy Approach for Multiple Sclerosis. J Nov Physiother. 2014;04(05).

36. Diechmann MD, Campbell E, Coulter E, Paul L, Dalgas U, Hvid LG. Effects of exercise training on neurotrophic factors and subsequent neuroprotection in persons with multiple sclerosis—a systematic review and meta-analysis. Brain Sci. 2021;11(11).

37. Sandroff BM, Jones CD, Baird JF, Motl RW. Systematic Review on Exercise Training as a Neuroplasticity-Inducing Behavior in Multiple Sclerosis. Neurorehabil Neural Repair. 2020;34(7):575–88.

38. Learmonth YC, Motl RW. Exercise Training for Multiple Sclerosis : A Narrative Review of History , Benefits , Safety , Guidelines , and Promotion. 2021;

39. Sun Y, Zehr EP. Training-induced neural plasticity and strength are amplified after stroke. Exerc Sport Sci Rev. 2019;47(4):223–9.

40. Garry MI, van Steenis RE, Summers JJ. Interlimb coordination following stroke. Hum Mov Sci. 2005;24(5–6):849–64.

41. Whitall J, McCombe Waller S, Sorkin JD, Forrester LW, Macko RF, Hanley DF, et al. Bilateral and unilateral arm training improve motor function through differing neuroplastic mechanisms: A single-blinded randomized controlled trial. Neurorehabil Neural Repair. 2011;25(2):118–29.

42. Smith AL, Richard Staines W. Cortical and behavioral adaptations in response to short-term inphase versus antiphase bimanual movement training. Exp Brain Res. 2010;205(4):465–77.

43. McCombe Waller S, Whitehall J. Bilateral arm training: Why and who benefits? NeuroRehabilitation. 2008;23:29–41.

44. Neva JL, Legon W, Staines WR. Primary motor cortex excitability is modulated with bimanual training. Neurosci Lett [Internet]. 2012;514(2):147–51. Available from: http://dx.doi.org/10.1016/j.neulet.2012.02.075

45. Stinear JW, Byblow WD. Disinhibition in the human motor cortex is enhanced by synchronous upper limb movements. J Physiol. 2002;543(1):307–16.

46. Liepert J, Mingers D, Heesen C, Bäumer T, Weiller C. Motor cortex excitability and fatigue in multiple sclerosis: A transcranial magnetic stimulation study. Mult Scler. 2005;11(3):316–21.

47. Toyokura M, Muro I, Komiya T, Obara M. Activation of pre-supplementary motor area (SMA) and SMA proper during unimanual and bimanual complex sequences: An analysis using functional magnetic resonance imaging. J Neuroimaging. 2002;12(2):172–8.

48. Staines WR, McIlroy WE, Graham SJ, Black SE. Bilateral movement enhances ipsilesional cortical activity in acute stroke: A pilot functional MRI study [4] (multiple

letters). Neurology. 2001;57(9):1740–1.

49. Calabrese M, Filippi M, Gallo P. Cortical lesions in multiple sclerosis. Nat Rev Neurol [Internet]. 2010;6(8):438–44. Available from: http://dx.doi.org/10.1038/nrneurol.2010.93

50. Kratochwill, T. R. Hitchcock, J. Horner, R. H. Levin, J. R. Odom, S. L. Rindskopf, D. M Shadish WR. Single-Case Design Technical Documentation. Work Clear website http//ies.ed.gov/ncee/wwc/pdf/wwc_scd.pdf. 2010;(December):2010.

51. Zhan S, Ottenbacher KJ. Single subject research designs for disability research. Disabil Rehabil. 2001;23(1):1–8.

52. Tate RL, Perdices M, Rosenkoetter U, Wakim D, Godbee K, Togher L, et al. Revision of a method quality rating scale for single-case experimental designs and n-of-1 trials: The 15-item Risk of Bias in N-of-1 Trials (RoBiNT) Scale. Neuropsychol Rehabil. 2013;23(5):619–38.

53. JF K. Rating neurologic impairment in multiple sclerosis: an expanded disability status scale (EDSS). Neurology. 1983;Nov;33(11):1444-52.

54. Meseguer-Henarejos AB, SăNCHEZ-MECA J, López-Pina JA, CARLES-HERNăNDEZ R. Inter-and intra-rater reliability of the Modified Ashworth Scale: A systematic review and meta-analysis. Eur J Phys Rehabil Med. 2018;54(4):576–90.

55. Sangarapillai K, Norman BM, Almeida QJ. Boxing vs Sensory Exercise for Parkinson's Disease: A Double-Blinded Randomized Controlled Trial. Neurorehabil Neural Repair. 2021;

56. Abbaspoor E, Zolfaghari M, Ahmadi B, Khodaei K. The effect of combined functional training on BDNF, IGF-1, and their association with health-related fitness in the multiple sclerosis women. Growth Horm IGF Res [Internet]. 2020;52(March):101320. Available from: https://doi.org/10.1016/j.ghir.2020.101320

57. DeLuca J, Chiaravalloti ND, Sandroff BM. Treatment and management of cognitive dysfunction in patients with multiple sclerosis. Nat Rev Neurol [Internet]. 2020;16(6):319–32. Available from: http://dx.doi.org/10.1038/s41582-020-0355-1

58. Kalb R, Brown TR, Coote S, Costello K, Dalgas U, Garmon E, et al. Exercise and lifestyle physical activity recommendations for people with multiple sclerosis throughout the disease course. Mult Scler J. 2020;26(12):1459–69.

59. Groppa S, Oliviero A, Eisen A, Quartarone A, Cohen LG, Mall V, et al. A practical guide to diagnostic transcranial magnetic stimulation: Report of an IFCN committee. Clin Neurophysiol [Internet]. 2012;123(5):858–82. Available from: http://dx.doi.org/10.1016/j.clinph.2012.01.010

60. Rossini PM, Burke D, Chen R, Cohen LG, Daskalakis Z, Di Iorio R, et al. Non-invasive electrical and magnetic stimulation of the brain, spinal cord, roots and peripheral nerves: Basic principles and procedures for routine clinical and research application: An updated report from an I.F.C.N. Committee. Clin Neurophysiol [Internet]. 2015;126(6):1071–107. Available from: http://dx.doi.org/10.1016/j.clinph.2015.02.001

61. Pascual-Leone A, Dang N, Cohen LG, Brasil-Neto JP, Cammarota A, Hallett M. Modulation of muscle responses evoked by transcranial magnetic stimulation during the acquisition of new fine motor skills. J Neurophysiol. 1995;74(3):1037–45.

62. Charalambous CC, Dean JC, Adkins DAL, Hanlon CA, Bowden MG. Characterizing the corticomotor connectivity of the bilateral ankle muscles during rest and isometric contraction in healthy adults. J Electromyogr Kinesiol [Internet]. 2018;41(February):9–18. Available from: https://doi.org/10.1016/j.jelekin.2018.04.009

63. Rossini PM, Barker AT, Berardelli A, Caramia MD, Caruso G, Cracco RQ, et al. Non-invasive electrical and magnetic stimulation of the brain, spinal cord, roots and peripheral nerves: Basic principles and procedures for routine clinical and research application: An updated report from an I.F.C.N. Committee. Clin Neurophysiol. 1994;91(2):79–92.

64. Awiszus F. TMS and threshold hunting [Internet]. Vol. 56. Elsevier B.V.; 2003. 13–23 p. Available from: http://dx.doi.org/10.1016/S1567-424X(09)70205-3

65. Silbert BI, Patterson HI, Pevcic DD, Windnagel KA, Thickbroom GW. Clinical Neurophysiology A comparison of relative-frequency and threshold-hunting methods to determine stimulus intensity in transcranial magnetic stimulation. Clin Neurophysiol [Internet]. 2013;124(4):708–12. Available from: http://dx.doi.org/10.1016/j.clinph.2012.09.018

66. Goldsworthy MR, Hordacre B, Ridding MC. Minimum number of trials required for within- and between-session reliability of TMS measures of corticospinal excitability. Neuroscience [Internet]. 2016;320:205–9. Available from: http://dx.doi.org/10.1016/j.neuroscience.2016.02.012

67. Snow NJ, Wadden KP, Chaves AR, Ploughman M. Review Article Transcranial Magnetic Stimulation as a Potential Biomarker in Multiple Sclerosis : A Systematic Review with Recommendations for Future Research. 2019;2019.

68. Franchignoni F, Horak F, Godi M, Nardone A, Giordano A. Using psychometric techniques to improve the balance evaluation systems test: The mini-bestest. J Rehabil Med. 2010;42(4):323–31.

69. Horak FB, Wrisley DM, Frank J. The balance evaluation systems test (BESTest) to differentiate balance deficits. Phys Ther. 2009;89(5):484–98.

70. Nieuwenhuis MM, Tongeren H Van, Sørensen PS, Ravnborg M. The Six Spot Step Test : a new measurement for walking ability in multiple sclerosis. 2006;(September 2005).

71. Callesen J, Richter C, Kristensen C, Sunesen I, Næsby M, Dalgas U, et al. Test–retest agreement and reliability of the Six Spot Step Test in persons with multiple sclerosis. Mult Scler J. 2019;25(2):286–94.

72. Carpinella I, Cattaneo D, Ferrarin M. Quantitative assessment of upper limb motor function in Multiple Sclerosis using an instrumented Action Research Arm Test. J Neuroeng Rehabil. 2014;11(1):1–16.

73. Andrews AW, Thomas MW, Bohannon RW. Normative values for isometric muscle force measurements obtained with hand-held dynamometers. Phys Ther. 1996;76(3):248–59.

74. Benedict RHB, Deluca J, Phillips G, LaRocca N, Hudson LD, Rudick R. Validity of the Symbol Digit Modalities Test as a cognition performance outcome measure for multiple sclerosis. Mult Scler. 2017;23(5):721–33.

75. Fisk JD, Ritvo PG, Ross L, Haase DA, Marrie TJ, Schlech WF. Measuring the functional impact of fatigue: Initial validation of the fatigue impact scale. Clin Infect Dis. 1994;18:S79–83.

76. Akbar N, Sandroff BM, Wylie GR, Strober LB, Smith A, Goverover Y, et al. Progressive resistance exercise training and changes in resting-state functional connectivity of the caudate in persons with multiple sclerosis and severe fatigue: A proof-of-concept study. Neuropsychol Rehabil [Internet]. 2020;30(1):54–66. Available from: https://doi.org/10.1080/09602011.2018.1449758

77. Lobo MA, Moeyaert M, Cunha AB, Babik I. Single-case design, analysis, and quality assessment for intervention research. J Neurol Phys Ther. 2017;41(3):187–97.

78. Ziemann U, Reis J, Schwenkreis P, Rosanova M, Strafella A, Badawy R, et al. TMS and drugs revisited 2014. Clin Neurophysiol [Internet]. 2015;126(10):1847–68. Available from: http://dx.doi.org/10.1016/j.clinph.2014.08.028

79. Hess CW, Mills KR, Murray NM. Responses in small hand muscles from magnetic stimulation of the human brain. J Physiol. 1987;388(1):397–419.

80. Vanteemar S. Sreeraj S, Uvais2 NA, Mohanty3 S, Kumar3 S, Department. Indian nursing students' attitudes toward mental illness and persons with mental illness. Ind Psychiatry J. 2019;195–201.

81. Paulus W, Peterchev A V., Ridding M. Transcranial electric and magnetic stimulation: Technique and paradigms. Handb Clin Neurol. 2013;116(0):329–42.

82. Balloff C, Penner I-K, Ma M, Georgiades I, Scala L, Troullinakis N, et al. The degree of cortical plasticity correlates with cognitive performance in patients with Multiple Sclerosis. Brain Stimul [Internet]. 2022;15(2):403–13. Available from: https://doi.org/10.1016/j.brs.2022.02.007

83. Stampanoni Bassi M, Buttari F, Maffei P, De Paolis N, Sancesario A, Gilio L, et al. Practice-dependent motor cortex plasticity is reduced in non-disabled multiple sclerosis patients. Clin Neurophysiol [Internet]. 2020;131(2):566–73. Available from: https://doi.org/10.1016/j.clinph.2019.10.023

84. Hallett M. Transcranial Magnetic Stimulation: A Primer. Neuron. 2007;55(2):187–99.

85. Zimnowodzki S, Butrum M, Kimura J, Stålberg E, Mahajan S, Gao L. Emergence of F-waves after repetitive nerve stimulation. Clin Neurophysiol Pract [Internet]. 2020;5:100–3. Available from: https://doi.org/10.1016/j.cnp.2020.04.002

86. Heyvaert M, Onghena P. Analysis of single-case data: Randomisation tests for measures of effect size. Neuropsychol Rehabil. 2014;24(3–4):507–27.

87. Krasny-Pacini A, Evans J. Single-case experimental designs to assess intervention effectiveness in rehabilitation: A practical guide. Ann Phys Rehabil Med [Internet]. 2018;61(3):164–79. Available from: http://dx.doi.org/10.1016/j.rehab.2017.12.002
